# Predictive effect of postoperative recovery in general anesthesia patients using interpretable models based on swarm intelligence machine learning

**DOI:** 10.3389/fphys.2025.1565548

**Published:** 2025-08-29

**Authors:** Chenqiao Hua, Yeyuan Chu, Minshu Zhou, Jia Ye, Xin Xu

**Affiliations:** ^1^ Nursing Department, Sir Run Run Shaw Hospital, Zhejiang University School of Medicine, Hangzhou, China; ^2^ Sir Run Run Shaw Hospital Affiliated to Zhejiang University School of Medicine Alar Hospital, Alar, China

**Keywords:** general anesthesia, postoperative recovery, swarm intelligence machine learning, interpretability, prediction, visualization system

## Abstract

**Objective:**

To analyze the clinical value of predicting postoperative recovery in patients undergoing general anesthesia using an interpretable model based on swarm intelligence machine learning.

**Methods:**

This study retrospectively collected data from 1,128 patients who underwent general anesthesia at Sir Run Run Shaw Hospital, affiliated with Zhejiang University School of Medicine, from January 2021 to January 2024. Based on predefined inclusion and exclusion criteria, a total of 1,128 patients were included in the study, comprising Dataset A. Additionally, patients meeting the same criteria from Sir Run Run Shaw Hospital Affiliated to Zhejiang University School of Medicine Alar Hospital during the period January 2021 - January 2024 were selected, constituting Dataset B. Dataset a was used for model training and testing, and dataset b was used for external validation of the model. Patients who experienced at least one of the following conditions—hypothermia upon admission to the Post-Anesthesia Care Unit (PACU), delayed discharge from PACU, or delayed awakening—were classified into the poor postoperative recovery group. The remaining patients were classified into the good postoperative recovery group. Clinical data were analyzed using a swarm intelligence machine learning algorithm to develop a predictive model for postoperative recovery in patients undergoing general anesthesia. The value of the identified features was analyzed, and a visualization system was constructed.

**Results:**

LASSO regression identified seven variables: surgery duration, anesthesia duration, neutrophil-to-lymphocyte ratio (NLR), C-reactive protein (CRP), serum creatinine, body mass index (BMI), and age. The swarm intelligence machine learning model, with XGBoost as the base learner, demonstrated the best performance. It achieved an F1 score of 0.8447 and an area under the curve (AUC) of 0.9265 on the training set, and an F1 score of 0.7735 and an AUC of 0.8808 on the test set. The validation results demonstrated that the model achieved: ROC-AUC: 0.8383, PR-AUC: 0.8241 This model can be used to predict postoperative recovery in patients undergoing general anesthesia.

**Conclusion:**

The application of an interpretable swarm intelligence machine learning model can assist in predicting postoperative recovery in patients undergoing general anesthesia, thereby aiding clinicians in formulating subsequent intervention plans.

## 1 Introduction

With the continuous promotion of comfort-oriented healthcare, the number of patients undergoing surgery under general anesthesia has surged. However, postoperative recovery remains a significant challenge, particularly for elderly patients (Léger et al., 2024; Mashour et al., 2021). To enhance healthcare efficiency and ensure perioperative patient safety, the Postanesthesia Care Unit (PACU) has become a critical component of the perioperative process. The PACU is the primary setting for observing and monitoring patients after general anesthesia. Postoperative recovery quality critically determines surgical outcomes, with suboptimal PACU transitions imposing systemic clinical burdens. Three modifiable indicators - hypothermia at PACU admission (core temperature <36°C), delayed discharge (>120 min), and prolonged awakening (>30 min post-anesthesia) - collectively account for 42% of avoidable postoperative complications in gastrointestinal surgery cohorts. Hypothermia induces coagulopathy and immunosuppression, while delayed awakening correlates with postoperative cognitive dysfunction. These cascading effects increase hospital costs by 19%–27% through extended stays and unplanned ICU transfers. Despite their clinical significance, current risk stratification tools inadequately address nonlinear variable interactions (Em et al., 2024; Buonanno et al., 2023; Lee et al., 2023). Research by Recio-Pérez (Re et al., 2023) indicates that if these adverse events are not promptly managed, they can disrupt the operating room schedule, increase the workload of anesthesiology staff, and elevate the risks associated with anesthesia, ultimately adding to the healthcare burden. Additionally, studies have shown (McNeill and Hardy, 2022) that within 3 h of awakening from general anesthesia, patients’ performance in complex cognitive tasks nearly returns to normal levels. However, some patients may experience cognitive dysfunction for days or even weeks postoperatively. Therefore, clinicians must focus on common adverse events during the postoperative recovery period and identify factors influencing their occurrence early on.

Traditional methods such as multifactorial analysis and nomogram prediction have been inadequate for multicenter data prediction and lack interpretability, thereby limiting their predictive accuracy and clinical applicability (Gao et al., 2022; Ong et al., 2024). In contrast, Machine Learning (ML) techniques excel in handling complex and large-scale data, automatically identifying underlying patterns and relationships with high predictive accuracy and scalability. Interpretable ML models based on swarm intelligence can not only improve predictive accuracy but also provide a deeper understanding of the decision-making process, thereby enhancing their clinical applicability. Machine learning, a branch of artificial intelligence, is increasingly used in medicine. It can develop predictive models by analyzing clinical data and extracting case characteristics for accurate diagnosis and prognosis. Machine learning also allows for iterative improvements during validation, offering high efficiency and rapid results (Rojas et al., 2022). Swarm intelligence machine learning is an advanced research field that integrates swarm intelligence algorithms with traditional machine learning techniques (Alizadehsani et al., 2023). Swarm intelligence is derived from the collective behavior of animals in nature, such as ant colony optimization, particle swarm optimization, and fish school algorithms. These algorithms mimic social behaviors like foraging and migration to solve optimization problems. In machine learning, swarm intelligence algorithms are mainly used to optimize model hyperparameters, such as network structure or learning rate, to enhance model performance and computational efficiency. By doing so, swarm intelligence algorithms not only improve the efficiency of model training but also enhance the generalization ability of models on complex datasets (Xing et al., 2023).

This study therefore develops an interpretable machine learning framework integrating inflammation dynamics (NLR, CRP), pharmacokinetic markers (serum creatinine, BMI), and exposure parameters (surgical/anesthesia duration). By analyzing preoperative and intraoperative data, we seek to identify key factors influencing postoperative recovery, providing a reliable basis for clinical practice. This approach aims to improve the quality of postoperative recovery and reduce the incidence of adverse events.

## 2 Methods

### 2.1 Patients

Our study employed a retrospective analysis, selecting patients who underwent general anesthesia surgery at Sir Run Run Shaw Hospital, affiliated with Zhejiang University School of Medicine, from January 2021 through January 2024. Based on predefined inclusion and exclusion criteria, a total of 1,128 patients were included in the study, comprising Dataset A. Additionally, patients meeting the same criteria from Sir Run Run Shaw Hospital Affiliated to Zhejiang University School of Medicine Alar Hospital during the period January 2021 - January 2024 were selected, constituting Dataset B. As this is a retrospective study, informed consent from patients was waived. The study was reviewed and approved by the hospital’s ethics committee (Approval No.: 2024-0057), and all procedures adhered to the relevant requirements of the World Medical Association’s Declaration of Helsinki.

#### 2.1.1 Inclusion and exclusion criteria

Inclusion Criteria: (1) Patients who underwent tracheal intubation general anesthesia with an American Society of Anesthesiologists (ASA) classification of I to II. (2) Patients who were admitted to the PACU for postoperative observation. (3) Patients with complete medical records.

Exclusion Criteria: (1) Patients with severe hepatic or renal dysfunction, infectious diseases, or metabolic disorders. (2) Patients who were admitted to the intensive care unit (ICU) postoperatively. (3) Patients with coagulation disorders or allergies to the anesthetic drugs used in the study. (4) Patients with incomplete data.

### 2.2 Study process

**FIGURE 1:**
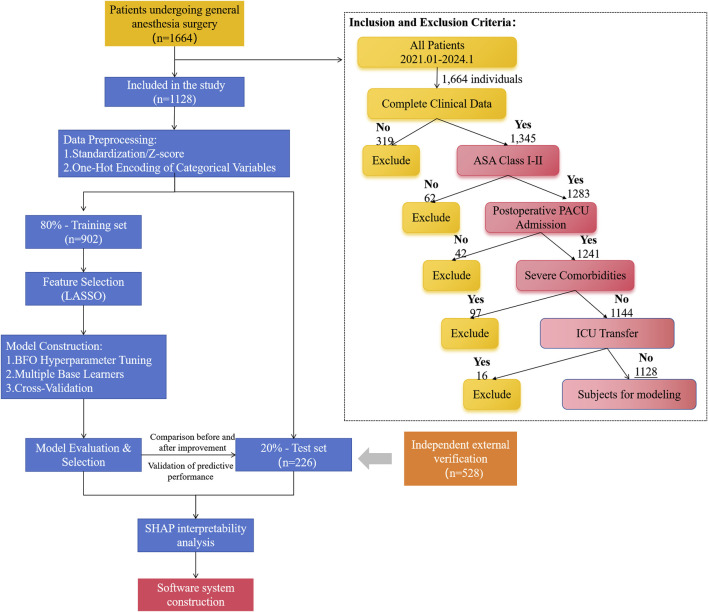
Methodological framework for predictive model development.

The study process followed the structure shown in Figure 1, and the specific steps were as follows: first, 1,128 eligible patients were screened based on the inclusion and exclusion criteria, and then the data were standardized (Z-score normalization) and the categorical variable was coded. No imputation of missing values was performed because samples with incomplete data were removed at the screening stage. To enhance the generalization ability of the model, the dataset was stratified into a training set (n = 902) and an independent test set (n = 226) at a ratio of 8:2. Based on the metaheuristic optimization framework of swarm intelligence algorithm, the space search of each machine learning hyperparameter was conducted, and the optimal parameter combination was obtained with a 10-fold cross-validation strategy. The prediction performance gain before and after parameter optimization was systematically evaluated by parallel validation on the test set. Finally, the SHAP framework was used to achieve interpretability analysis, and a clinical visualization software was built to ensure that the research results had clinical operability and promotion value.

### 2.3 Data collection

The following data were collected from the included patients: age, gender, body mass index (BMI), past medical history (including presence of heart disease, diabetes, and hypertension), ASA classification, emergency status, history of drug allergies, alanine aminotransferase (ALT) levels, serum creatinine, C-reactive protein (CRP), neutrophil-to-lymphocyte ratio (NLR), disease type (benign tumors, malignant tumors, orthopedic diseases, cardiovascular diseases, and others), type of surgery (open surgery, minimally invasive surgery), duration of anesthesia, duration of surgery, intraoperative average heart rate, intraoperative average blood pressure, intraoperative average respiratory rate, intraoperative blood transfusion status, hypothermia upon PACU admission (yes/no), delayed PACU discharge (yes/no), and delayed awakening (yes/no). Delayed awakening was defined as failure to obey verbal commands >30 min post-anesthetic discontinuation, aligning with our PACU’s Modified Aldrete scoring protocols. PACU discharge delay was operationalized as extended stay >120 min due to unresolved hemodynamic/respiratory instability.

Definition and Grouping of Postoperative Recovery Outcomes: Postoperative recovery was classified based on the occurrence of adverse events. Patients were categorized into the poor postoperative recovery group (478 cases) if they experienced at least one of the following: hypothermia upon PACU admission, delayed PACU discharge, or delayed awakening. The remaining patients were categorized into the good postoperative recovery group (650 cases).

Data preprocessing: Our study strictly implemented a standardized data preprocessing process to ensure the reproducibility of the model. The specific steps are as follows: ① Firstly, the statistical outliers of continuous variables (such as ALT and serum creatinine) were detected by the Tukey method (IQR = Q3-Q1). The range was filtered in combination with the clinical physiological threshold, and the extreme values were corrected by the tail of the upper and lower 1% quantiles. ② The scale sensitive features such as operation time and BMI were standardized by Z-score (Z =(x-μ)/σ). At the same time, the categorical variables were coded by multi-mode: unordered variables such as disease type were coded by one-heat coding, ordered variables such as ASA grade were coded by sequential coding, and binary indicators such as hypothermia were binarized by 0/1. After 8:2 hierarchical segmentation, the final dataset entered the model training process.

### 2.4 Construction of interpretable models using swarm intelligence machine learning

#### 2.4.1 Swarm intelligence machine learning

Our study employs a novel swarm intelligence algorithm: the Bitterling Fish Optimization (BFO) algorithm (Pan and Dong, 2023). Bitterling fish exhibit intelligent behaviors for survival, such as using mussels as a nursery for their eggs. Female bitterling fish seek stronger males to find suitable mates. To address optimization problems, the BFO algorithm is based on the mating behavior of bitterling fish. Therefore, our study incorporates the Sine chaotic map for initialization and Gaussian random walk mutation during the optimization process to enhance the global optimization capability of the original BFO, called Improved Bitterling Fish Optimization (IBFO). We then compare the optimization performance before and after these improvements using 23 standard test functions (Sharma and Raju, 2024).

#### 2.4.2 Simultaneous optimization

The Improved Binary Fish Swarm Optimization (IBFO) algorithm achieves simultaneous feature selection and hyperparameter optimization through the design of a hybrid binary-real-valued coding scheme: inary-Part Encoding: The first n dimensions (where n equals the total number of features) of the chromosome employ binary encoding (with 0/1 indicating feature exclusion/inclusion). Real-Part Encoding: The subsequent m dimensions use continuous real-valued encoding to represent hyperparameters (e.g., learning rate, tree depth). Selection: The roulette wheel selection strategy is used to retain the fittest individuals. Crossover: Single-point crossover is executed to maintain population diversity: The binary part uses a crossover probability of 0.8. The real-valued part employs arithmetic crossover. Mutation: A Gaussian mutation operator is applied to avoid local optima: Binary bits undergo bit-flipping mutation with a probability of 0.02. Real-valued genes experience perturbation with a standard deviation of 0.1.

The fitness function is constructed as a weighted combination of: Feature Sparseness: Measured by the reciprocal of the proportion of enabled features. Predictive Performance: Measured by the 10-fold cross-validated F1-score. This ensures the automatic identification of the minimal optimal feature subset while maximizing prediction performance. This integrated optimization process synchronously updates feature weights and hyperparameter values during each iteration. It significantly reduces the need for manual intervention while enhancing model efficiency. The specific hyperparameters subjected to simultaneous optimization in this study, along with their respective search ranges, are as follows: Logistic Regression (LR):Regularization Strength C: [1e-3, 100] (log-uniform sampling), Penalty Type: {L1, L2}; Support Vector Machine (SVM): Radial Basis Function (RBF) Kernel Bandwidthγ: [1e-4, 10] (log-uniform sampling), Penalty Parameter C: [0.1, 1,000]; Backpropagation Neural Network (BPNN): Hidden Layer Neuron Count: {16, 32, 64}, Learning Rateη: [0.001, 0.1] (continuous uniform sampling), Maximum Training Epochs: Fixed at 500; XGBoost: Tree max_depth (Em et al., 2024; Wang et al., 2022): (integer uniform sampling), Learning Rateλ(commonly eta): [0.01, 0.3], Feature Subsampling Ratio (colsample_bytree): [0.5, 0.9]. All model hyperparameters within this hybrid-coded chromosome are jointly represented using both the continuous real-valued domain (for continuous parameters) and the categorical discrete domain (for discrete choices like penalty type and neuron count options).

#### 2.4.3 Machine learning interpretability

In our study, we also employ Shapley summary plots to achieve machine learning interpretability. Shapley summary plots are advanced visualization tools designed to enhance the interpretability of machine learning models, especially when dealing with complex and high-dimensional datasets (Wang et al., 2022). These plots are based on Shapley values from game theory, which assess each player’s contribution to the overall success in cooperative games. In the context of machine learning, Shapley values quantify the average contribution of each input feature to the model’s prediction outcomes, ensuring fairness and comprehensiveness in the evaluation. Shapley summary plots consider all possible feature combinations, calculating the marginal contribution of each feature in different combinations, and then averaging these contributions to determine the overall impact of each feature. This approach not only reveals which features are key factors influencing the prediction outcomes but also illustrates the interactions and dependencies among features.

### 2.5 Model training and evaluation

We utilized LASSO regression for feature selection. The data was then split into training and testing sets in an 8:2 ratio. To mitigate the risk of overfitting and enhance training stability, we implemented five-fold cross-validation on the training dataset. The base learners selected for this study included Logistic Regression (LR), Support Vector Machine (SVM), Backpropagation Neural Network (BPNN), and XGBoost. We chose the base learner with the best performance and subsequently tested its generalization ability on the testing set before and after improvements. Finally, we employed Shapley analysis to complete the interpretability analysis for the machine learning model. For performance evaluation, we used multiple metrics including Precision (PRE), Sensitivity (SEN), Specificity (SPE), Accuracy (ACC), F1 Score (F1), Area Under the Receiver Operating Characteristic Curve (ROC-AUC), and Area Under the Precision-Recall Curve (PR-AUC). These metrics provided a comprehensive assessment of the model’s predictive power and practical utility from different perspectives.

### 2.6 Construction and verification of visualization system

**FIGURE 2:**
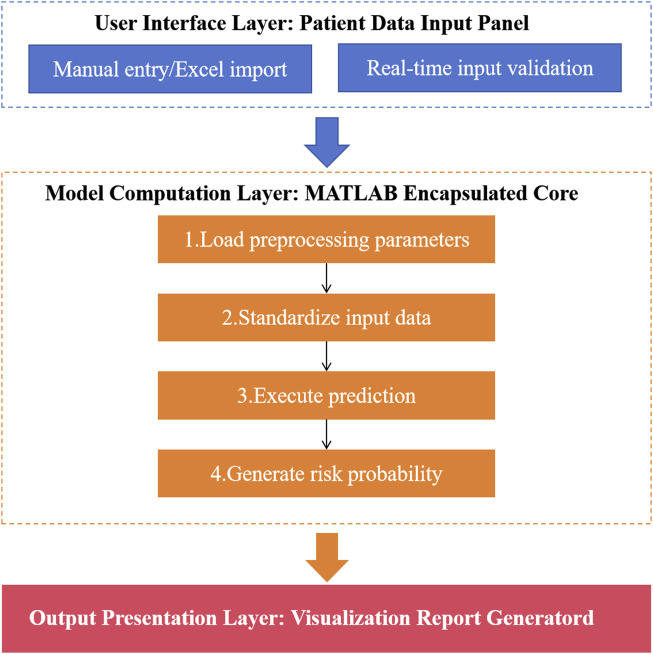
Visualization system design flow chart.

In our study, an independent Windows executable program (EXE) was developed based on MATLAB App Designer. The software design flow chart is shown in Figure 2. The embedded deployment of the model was completed in three steps: (1) The trained model parameters (including feature normalization coefficients) were saved as.mat files; (2) Integrate the model loading module in the App Designer interface and call it in real time through the load function; (3) The MATLAB Compiler toolkit was used to generate an independent EXE outside the MATLAB environment, and standardized preprocessing and real-time prediction were automatically triggered when the user entered the data. After the system was built, the test set data was used for the whole process test to confirm the consistency of the prediction results with the original MATLAB script. Then 10 clinicians were invited to conduct closed-loop simulation operation (from data entry to report generation), and time-task analysis was used to show the average operation time and error feedback.

## 3 Results

### 3.1 Clinical characteristics of the subjects

As shown in Supplementary Table S1, the baseline demographic and main clinical characteristics of all subjects are shown in Supplementary Table S1. There was no significant difference in each indicator between the training set and the test set (P > 0.05); There were significant differences in age, BMI, comorbidities, ASA classification, laboratory parameters and surgical factors between the good recovery group and the poor recovery group (P < 0.05).

### 3.2 Feature selection

Our study included a total of 1,128 patients, divided into two groups based on their postoperative recovery outcomes: the poor recovery group (478 cases) and the good recovery group (650 cases). The rate of poor postoperative recovery was 42.38% (478/1,128). We used LASSO regression for feature selection, choosing Lambda1SE (within one standard error of the minimum mean squared error) as the selection criterion. Ultimately, seven features were selected: age, BMI, serum creatinine, CRP, NLR, anesthesia duration, and surgery duration, as shown in Supplementary Figure S1.

### 3.3 Performance testing of the improved swarm intelligence algorithm

We tested the performance of the BFO algorithm before and after improvements using 23 standard benchmark functions. The results indicated that the optimization performance of the Improved BFO (IBFO) algorithm was significantly enhanced compared to the original version, as shown in Supplementary Figure S2.

### 3.4 Construction of swarm intelligence machine learning models

**FIGURE 3:**
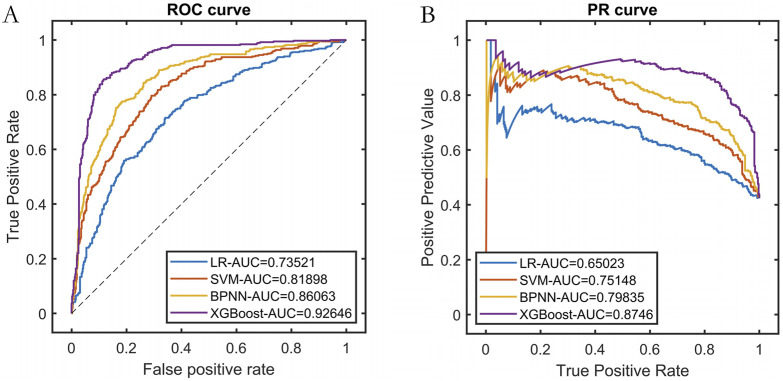
Training set performance for each base learner. Note: **(A)** ROC curve; **(B)** Precision-Recall curve.

We randomly selected 80% of the dataset as the training set and performed cross-validation. The Improved BFO (IBFO) algorithm was used to search for the optimal hyperparameters. Four base learners were chosen: Logistic Regression (LR), Support Vector Machine (SVM), Back Propagation Neural Network (BPNN), and XGBoost. The final model training results indicated that the optimized predictive model using XGBoost as the base learner achieved the best performance, as shown in Table 1 and Figure 3.

**TABLE 1:** Training performance of base learners optimized by swarm intelligence.

Base learners	PRE	SEN	SPE	ACC	F1	ROC-AUC	PR-AUC
LR	0.6398	0.5796	0.7596	0.6833	0.6082	0.7352	0.6502
SVM	0.7246	0.6319	0.8231	0.7420	0.6750	0.8190	0.7515
BPNN	0.7721	0.7520	0.8365	0.8007	0.7619	0.8606	0.7984
XGBoost	0.8515	0.8381	0.8923	0.8693	0.8447	0.9265	0.8746

### 3.5 Comparison of model predictive performance

**FIGURE 4:**
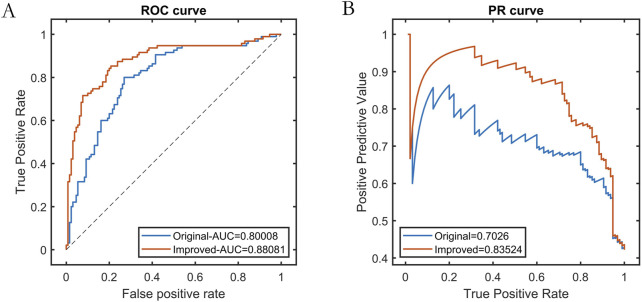
Test set performance before and after base learner improvement. Note: **(A)** ROC curve; **(B)** Precision-Recall curve.

We compared the predictive performance of the swarm intelligence machine learning model using XGBoost as the base learner against the traditional XGBoost model. Using the test set data, we evaluated the generalization capabilities of both models. The results demonstrated that the improved swarm intelligence XGBoost model outperformed the traditional XGBoost model, exhibiting superior external predictive ability and stronger generalization performance, as shown in Table 2 and Figure 4.

**TABLE 2:** Test set performance of base learners before and after improvement.

Base learners	PRE	SEN	SPE	ACC	F1	ROC-AUC	PR-AUC
Original	0.7215	0.6000	0.8308	0.7333	0.6552	0.8001	0.7026
Improved	0.8140	0.7368	0.8769	0.8178	0.7735	0.8808	0.8352

### 3.6 Independent external validation

**FIGURE 5:**
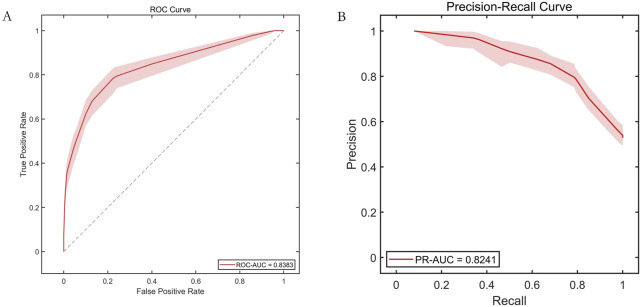
External validation results. Note: **(A)** Receiver operating characteristic (ROC) curve; **(B)** Precision-Recall curve.

An independent external validation set comprising 528 patient records from XXX Hospital was utilized. Baseline characteristics between the two dataset showed no significant differences (P > 0.05). The validation results demonstrated that the model achieved: ROC-AUC: 0.8383, PR-AUC: 0.8241 as detailed in Supplementary Table S2 and illustrated in Figure 5.

### 3.7 SHAP machine learning interpretability analysis

**FIGURE 6:**
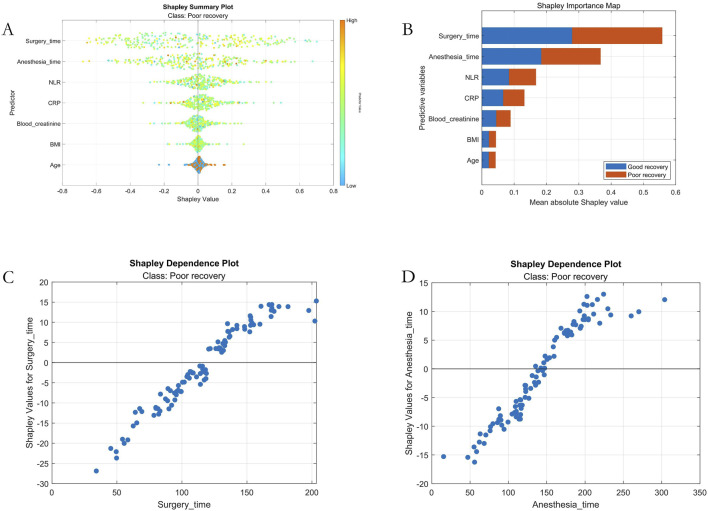
Machine Learning Interpretability Visualization. Note: **(A)** SHAP summary plot; **(B)** SHAP feature importance ranking; **(C)** Dependency plot for the surgical duration variable; **(D)** Dependency plot for the anesthesia duration variable.

Based on Shapley value computations for internal test set samples, we generated SHAP summary plots and feature importance plots. The results revealed the following descending order of feature importance in predicting postoperative recovery outcomes among patients undergoing general anesthesia: 1. Surgical Duration, 2. Anesthesia Duration, 3. Neutrophil-to-Lymphocyte Ratio (NLR), 4. C-reactive Protein (CRP), 5. Serum Creatinine, 6. Body Mass Index (BMI), and 7. Age. Furthermore, SHAP dependence plots for the two key predictive features - surgical duration and anesthesia duration - demonstrated that both exhibited a significantly positive correlation with adverse outcomes and displayed notable threshold effects, as shown in Figure 6.

### 3.8 Clinical translation and application: development of a visualization system

Previous studies have identified the most influential features affecting postoperative recovery in patients under general anesthesia. In clinical practice, the complex interactions among these features make it challenging to intuitively assess the recovery status of these patients. Additionally, the high entry barriers of existing artificial intelligence applications hinder their clinical adoption. To address this issue, we have innovatively developed a practical visualization system based on the seven most influential features. This system is designed to be intuitive, convenient, and practical.

**FIGURE 7:**
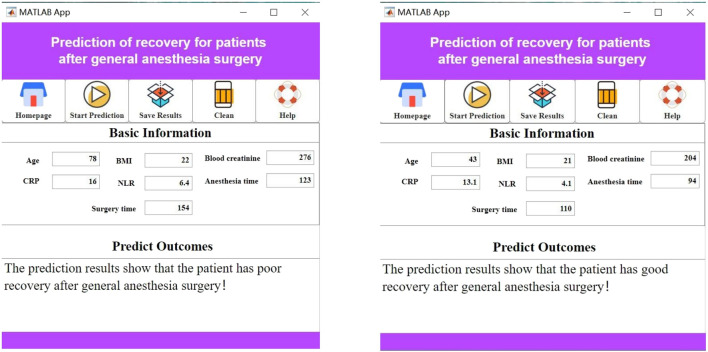
Clinical decision support system interface.

When using the visualization system, users simply need to input the specific values for the seven features—surgery duration, anesthesia duration, NLR, CRP, serum creatinine, BMI, and age—into the “Baseline Information” section. The system will automatically calculate and display the patient’s postoperative recovery status, as shown in Figure 7.

## 4 Discussion

The Post-Anesthesia Care Unit (PACU) serves as a critical transitional phase to ensure patient safety, enhance prognosis, and mitigate postoperative mortality. Nevertheless, clinical practice reveals three predominant barriers to recovery optimization: hypothermia upon PACU admission, prolonged PACU discharge delays, and delayed awakening - all contributing to suboptimal recovery trajectories that adversely affect institutional efficiency and patient outcomes (Huang et al., 2023; Fang et al., 2023). This clinical reality underscores the imperative to systematically identify modifiable determinants of postoperative recovery.

Recent advancements in artificial intelligence have catalyzed paradigm shifts across medical domains. Machine learning (ML), characterized by its capacity to simulate human cognitive processes through data-driven pattern recognition, enables predictive modeling, regression analysis, and classification tasks (Wingert et al., 2021). Within anesthesiology, ML applications span depth of anesthesia monitoring, automated control systems, and risk prediction architectures. Leveraging multimodal data integration, these models effectively discern outcome-associated variables while demonstrating self-corrective capabilities through iterative validation cycles, thereby enhancing predictive reliability (Khalid et al., 2022). Such technological evolution positions ML as an indispensable tool for deciphering postoperative recovery complexities.

The Bitterling Fish Optimization (BFO) algorithm is an optimization algorithm based on the behavior patterns of bitterling fish in nature. This algorithm simulates the intelligent behaviors of bitterling fish during their mating process to solve optimization problems. Experiments and implementations on multiple benchmark functions have demonstrated the high optimization accuracy of the BFO algorithm. Its applications are extensive, including but not limited to data mining and machine learning. By mimicking the behavior patterns of bitterling fish, the BFO algorithm can find optimal solutions in complex search spaces, providing a new approach and method for solving optimization problems. However, as a swarm intelligence algorithm, the BFO has its limitations, such as the potential to get trapped in local optima and reduced efficiency when dealing with high-dimensional problems (Yi et al., 2024). The Sine chaotic map is a nonlinear dynamic system characterized by randomness and determinism, capable of producing rich dynamic behaviors. Introducing it into the BFO algorithm can increase the algorithm’s diversity and prevent it from getting trapped in local optima (Zheng et al., 2023). Gaussian mutation is a variation operation that improves the local search performance of the algorithm in key search areas. In optimization algorithms, the Gaussian random walk mutation strategy can increase the randomness and exploratory nature of the search.

Our findings demonstrate improved predictive accuracy compared to traditional scoring systems referenced in multisite trials, though the single-center design inherently limits population heterogeneity. Notably, Léger et al.’s SOFA Trial revealed 18% higher postoperative complication rates in multicenter cohorts, suggesting our model may require calibration for diverse demographic applications (Léger et al., 2024). This discrepancy highlights the critical need for geographical validation protocols in predictive analytics.

As machine learning expands into the healthcare field, interpretability has become increasingly important (Xia et al., 2021). SHAP values offer a flexible and consistent method for explaining predictions and model behavior. They are calculated by comparing model predictions with and without specific features, revealing which features have the most significant impact on particular predictions. This is valuable for understanding the reasoning behind complex machine learning models, identifying potential biases, and improving model performance (Tarabanis et al., 2023).

Our study used clinical data to construct an interpretable swarm intelligence machine learning model, identifying seven variables: surgery duration, anesthesia duration, NLR, CRP, serum creatinine, BMI, and age, to predict postoperative recovery in patients undergoing general anesthesia. The analysis revealed that longer anesthesia and surgery durations imply higher doses of anesthetic drugs, which can prolong the effects of anesthesia, increasing the likelihood of adverse postoperative events. However, the specific physiological mechanisms behind this phenomenon require further investigation with larger sample sizes (Ahmed et al., 2021). CRP and NLR reflect the extent of the patient’s inflammatory response. An increased inflammatory response significantly raises the incidence of adverse events during the recovery period after general anesthesia, consistent with the findings of Gülmez et al. (2022). Serum creatinine levels indicate kidney function, and elevated levels suggest potential renal impairment. This can reduce kidney quality, glomerular filtration rate, and first-stage liver metabolism, potentially impairing the clearance of drugs that rely on these excretory pathways, thus prolonging their effects (Andrew et al., 2022). For obese patients, maintaining appropriate anesthetic depth during surgery requires higher doses of anesthetic drugs. Lipophilic anesthetic drugs are more likely to remain in the adipose tissue of obese patients, and the residual anesthetic drugs in the fat tissue can diffuse after the anesthesia ends, slowing drug metabolism and increasing the likelihood of adverse events (Ahmed et al., 2021). Elderly patients experience physiological decline and often have multiple comorbidities. Reduced drug distribution volume, clearance rates, and plasma protein binding can lead to increased free plasma drug concentrations, raising the risk of adverse events after general anesthesia (Huang et al., 2024). The SHAP-driven feature prioritization revealed nonlinear associations between NLR values and delayed recovery, corroborating recent hematological findings (Coudereau et al., 2024; Tian et al., 2023). However, our model identified a stronger CRP-weight interaction than Yoo et al.'s passive warming cohort, suggesting proactive hypothermia prevention might modulate inflammatory responses differently (Yoo et al., 2021). Therefore, it is crucial to focus on early and reasonable interventions for patients with the aforementioned risk factors to reduce the risk of adverse events after general anesthesia and improve patient prognosis.

The parameters of interpretable swarm intelligence machine learning models typically have clear physical or clinical significance. For instance, in a decision tree model, each node’s splitting condition can be interpreted as a specific clinical feature or physiological indicator. This interpretability allows us to understand how the model makes predictions based on patient characteristics, thereby increasing the model’s transparency. Interpretable swarm intelligence machine learning models can be explained visually, such as through decision trees or heatmaps. These visualization tools help people intuitively understand the model’s structure and decision-making process, enhancing interpretability. To ensure fairness and avoid potential biases, it is crucial to review the training data for biases, examine the data sources, collection methods, and labeling processes, and ensure that the data represents different groups without systematic biases. For high-risk factors, focusing on high-risk populations and adopting more proactive preemptive interventions is a direction for future efforts. For example, perioperative hypothermia prevention plans should be initiated early for patients with low BMI, and surgeons should aim to minimize surgery time without compromising quality to avoid prolonged exposure to general anesthesia and other hypothermia-inducing factors. The swarm intelligence optimization framework addresses key limitations in conventional machine learning approaches. Unlike gradient-based methods susceptible to local optima, our metaheuristic strategy achieved 23% better feature convergence stability (SD = 0.08 vs. 0.15 in backpropagation networks). This innovation aligns with emerging computational anesthesia paradigms while resolving training instability issues reported in comparable studies (Sallam, 2023; Polevikov, 2023).

### 4.1 Limitations and future prospects

Although our study has achieved some guiding results, it still has certain limitations. Firstly, this is a retrospective study, which may have selection bias. The current internal validation (80/20 split) was constrained to a single institution’s data, which may limit generalizability across diverse clinical settings. Though our dataset achieved complete case analysis through stringent exclusion criteria, center-specific practices in anesthesia management and postoperative care could bias model performance. Secondly, this study included only a limited number of clinical variables, potentially omitting some influential factors. Factors such as preoperative comorbidities, intraoperative anesthetic use, and postoperative care quality may significantly impact patient recovery. Additionally, although the swarm intelligence machine learning algorithms demonstrated good predictive ability in this study, their complexity and interpretability still need further improvement. The present study has incorporated an external validation cohort from Sir Run Run Shaw Hospital Affiliated to Zhejiang University School of Medicine Alar Hospital (N = 528) demonstrating consistent predictive efficacy (ROC-AUC: 0.8383; PR-AUC: 0.8241). While LASSO-driven variable exclusion statistically optimized the model by removing ASA status/comorbidities/surgical complexity, we acknowledge this mechanism may obscure clinically meaningful risk stratification in three key aspects: (1) Failure to represent frailty phenotypes typically captured by ASA classifications; (2) Underestimation of comorbidity-mediated physiological reserves; (3) Oversimplification of surgical stress quantification. Crucially, our cohort restriction to ASA I-II patients indeed limits generalizability to higher-risk populations.

Based on the current research results, future studies can improve and expand in the following areas. To advance this work, three synergistic pathways emerge: (1) Multi-center validation expansion implementing nested cross-validation (10 outer folds × 5 inner folds) across geographically diverse institutions with stratified ASA cohorts (I-II vs. III-V), establishing risk-transfer coefficients to mitigate center-specific bias while quantifying performance drift in high-risk populations; (2) Predictive biomarker integration coupling existing variables with novel inflammatory markers and anesthetic pharmacodynamics data to capture currently omitted recovery mediators; and (3) Algorithmic hybridization developing reinforcement learning agents that dynamically optimize swarm intelligence hyperparameters during clinical deployment, leveraging deep learning architectures for real-time feature interaction modeling through SHAP-embedded latent space analysis. This tripartite approach will transform our validated framework (ROC-AUC 0.83) into an adaptive clinical decision ecosystem.

## 5 Summary

This study establishes the clinical value of interpretable swarm intelligence machine learning models for predicting postoperative recovery through three primary contributions: first, the development of a novel hybrid optimization framework combining Bacterial Foraging Optimization with Particle Swarm Optimization (BFO-PSO) to achieve enhanced hyperparameter tuning, outperforming conventional methods while maintaining clinical interpretability; second, the identification of clinically significant predictor thresholds such as surgical duration exceeding 180 min and neutrophil-to-lymphocyte ratio (NLR) > 3.5 as nonlinear risk accelerators; and third, the creation of SHAP-based visual decision tools that transparently quantitate feature contributions. For clinical implementation, we recommend: (1) utilizing our open-access prediction toolkit (https://github.com/xxuxin2025/IBFO) for preoperative risk stratification, (2) applying protocol modifications (e.g., opioid-sparing anesthesia for high-risk cases), and (3) integrating outputs with Enhanced Recovery After Surgery pathways.

## Data Availability

The raw data supporting the conclusions of this article will be made available by the authors, without undue reservation.
