# Supplementary material for: Predictive effect of postoperative recovery in general anesthesia patients using interpretable models based on swarm intelligence machine learning
Source: Front Physiol. 2025 Aug 29;16:1565548. doi: 10.3389/fphys.2025.1565548 (PMC12426152; doi:10.3389/fphys.2025.1565548)
Supplement: Supplementary file 1 [file Table1.docx]

****Supplementary Table 1. Baseline Characteristics of Study Participants****

| Variables | Dataset Group | | Outcome Group | |
| --- | --- | --- | --- | --- |
|  | Training Set (n=902) | Test Set (n=226) | Good Recovery (n=650) | Poor Recovery (n=478) |
| **Demographics** |  |  |  |  |
| Age (years) | 55.89±11.93 | 57.84±12.64 | 53.17±10.24* | 60.75±12.89* |
| BMI (kg/m²) | 24.12±4.15 | 23.91±4.67 | 23.41±3.85* | 24.99±4.58* |
| Male, n (%) | 492 (54.55%) | 120 (53.10%) | 342 (52.62%) | 270 (56.49%) |
| **Comorbidities** |  |  |  |  |
| Cardiovascular disease, n (%) | 214 (23.73%) | 51 (22.57%) | 120 (18.46%)* | 145 (30.33%)* |
| Diabetes mellitus, n (%) | 141 (15.63%) | 32 (14.16%) | 85 (13.08%)* | 88 (18.41%)* |
| Hypertension, n (%) | 322 (35.70%) | 76 (33.63%) | 190 (29.23%)* | 208 (43.51%)* |
| **ASA Classification** |  |  |  |  |
| ASA I, n (%) | 320 (35.48%) | 75 (33.19%) | 280 (43.08%)* | 115 (24.06%)* |
| ASA II, n (%) | 582 (64.52%) | 151 (66.81%) | 370 (56.92%)* | 363 (75.94%)* |
| **Laboratory Parameters** |  |  |  |  |
| ALT (U/L) | 28.93±13.76 | 30.12±15.01 | 26.31±12.05* | 33.06±15.69* |
| Serum creatinine (μmol/L) | 76.95±17.92 | 78.23±19.01 | 72.15±15.43* | 84.08±18.92* |
| CRP (mg/L) | 8.12±5.03 | 8.51±5.42 | 6.83±4.12* | 10.06±5.87* |
| NLR | 3.79±2.05 | 3.94±2.27 | 3.42±1.88* | 4.36±2.37* |
| **Surgical Factors** |  |  |  |  |
| Anesthesia duration (min) | 143.15±47.82 | 148.63±50.11 | 128.45±41.23* | 165.73±50.89* |
| Operative duration (min) | 121.34±43.05 | 124.95±46.32 | 110.25±38.71* | 138.54±46.85* |
| Intraoperative transfusion, n (%) | 226 (25.06%) | 52 (23.01%) | 112 (17.23%)* | 166 (34.73%)* |

**Note:** * indicates p<0.05 compared with the Good Recovery group.
