# Supplementary figures and images for: Predictive effect of postoperative recovery in general anesthesia patients using interpretable models based on swarm intelligence machine learning
Source: Front Physiol. 2025 Aug 29;16:1565548. doi: 10.3389/fphys.2025.1565548 (PMC12426152; doi:10.3389/fphys.2025.1565548)

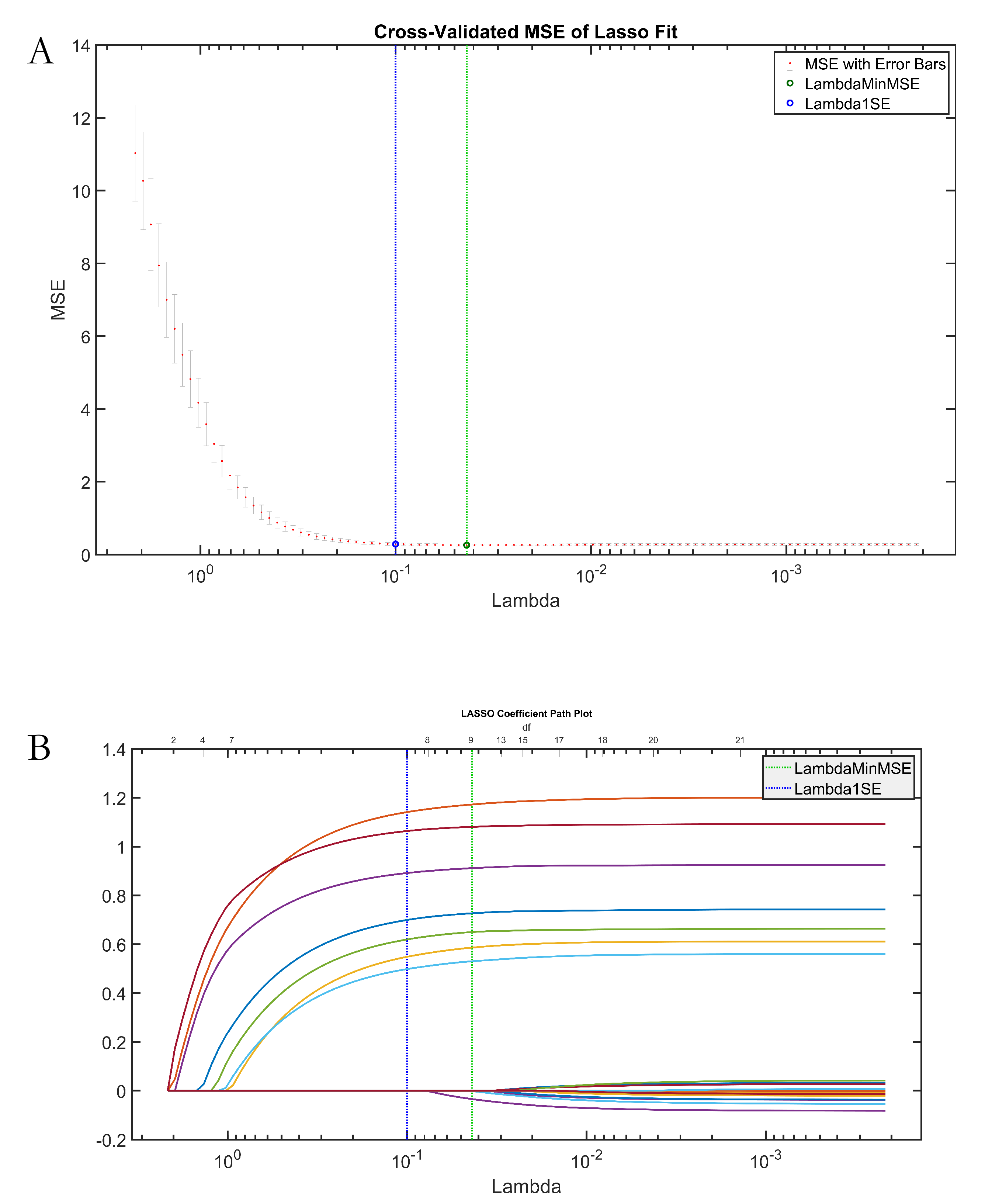

Supplement: Supplementary file 2 [file Image1.jpeg]

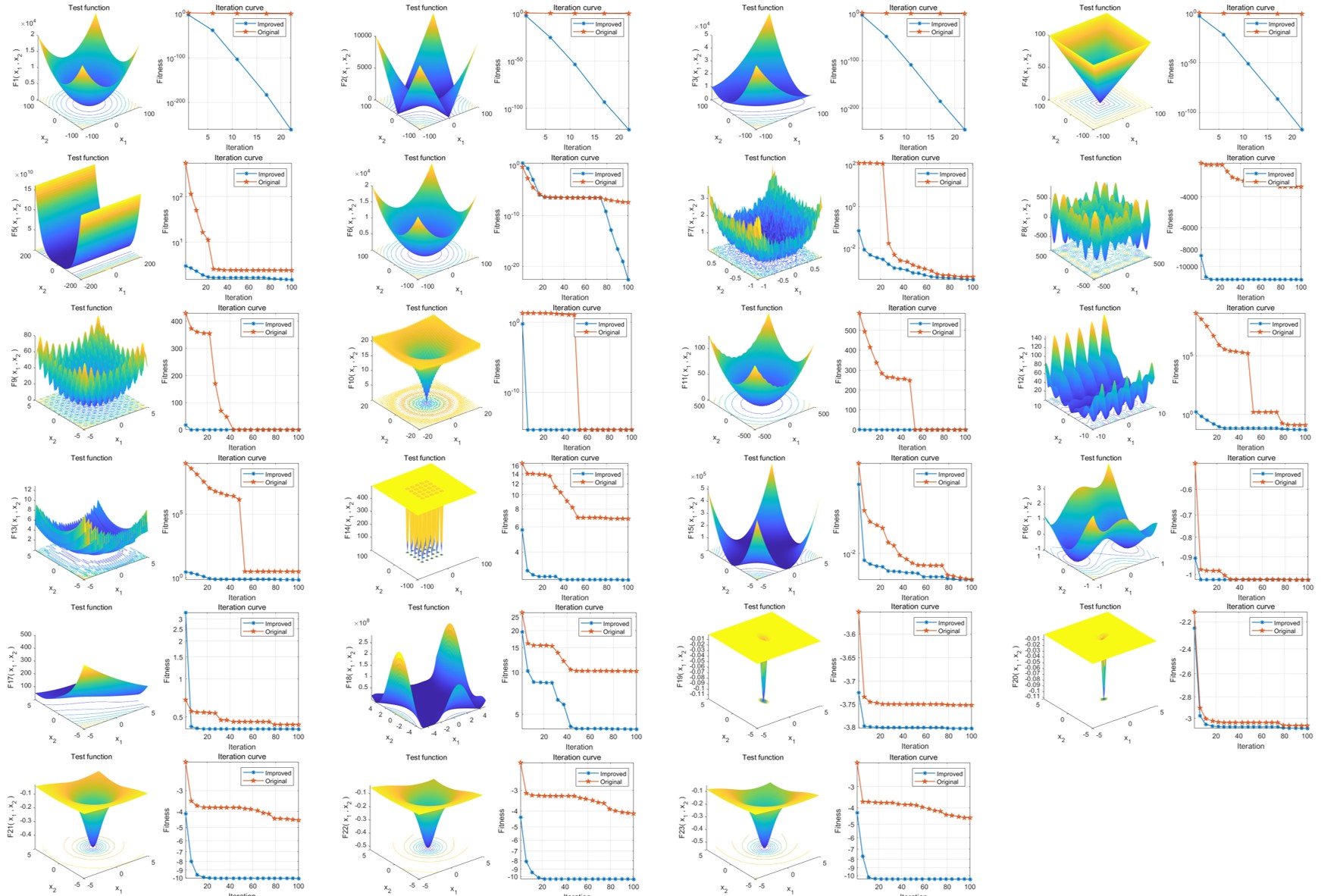

Supplement: Supplementary file 3 [file Image2.jpeg]
