# Supplementary material for: Predictive effect of postoperative recovery in general anesthesia patients using interpretable models based on swarm intelligence machine learning
Source: Front Physiol. 2025 Aug 29;16:1565548. doi: 10.3389/fphys.2025.1565548 (PMC12426152; doi:10.3389/fphys.2025.1565548)
Supplement: Supplementary file 4 [file Table2.docx]

**Supplementary Table 2 Baseline data were compared between the two dataset**

| Variable | Dataset A (n=1128) | Dataset B (n=528) | Statistic | p-value |
| --- | --- | --- | --- | --- |
| **Demographics** |  |  |  |  |
| Age (years), mean±SD | 56.28±12.11 | 56.95±11.83 | t=1.058 | 0.291 |
| BMI (kg/m²), mean±SD | 24.08±4.28 | 24.22±4.35 | t=0.617 | 0.537 |
| Male, n (%) | 612 (54.26%) | 321 (60.80%) | χ²=6.254 | **0.012** |
| **Comorbidities** |  |  |  |  |
| Cardiovascular disease, n (%) | 265 (23.49%) | 127 (24.05%) | χ²=0.062 | 0.803 |
| Diabetes mellitus, n (%) | 173 (15.34%) | 86(16.29%) | χ²=0.247 | 0.620 |
| Hypertension, n (%) | 398 (35.28%) | 187 (35.42%) | χ²=0.003 | 0.958 |
| **ASA Classification** |  |  | χ²=0.005 | 0.946 |
| ASA I, n (%) | 395 (35.02%) | 184 (34.85%) |  |  |
| ASA II, n (%) | 733 (64.98%) | 344 (65.15%) |  |  |
| **Laboratory Parameters** |  |  |  |  |
| Serum creatinine (μmol/L), mean±SD | 77.18±17.62 | 76.83±17.91 | t=0.375 | 0.708 |
| CRP (mg/L), mean±SD | 8.17±5.19 | 8.39±5.24 | t=0.801 | 0.423 |
| NLR, mean±SD | 3.81±2.14 | 3.87±2.08 | t=0.537 | 0.592 |
| **Surgical Factors** |  |  |  |  |
| Anesthesia duration (min), mean±SD | 144.12±48.05 | 142.86±47.92 | t=0.498 | 0.619 |
| Operative duration (min), mean±SD | 121.87±43.49 | 120.35±43.87 | t=0.661 | 0.509 |
